# Molecular Prevalence of Hemotropic Mycoplasmosis and Associated Risk Factors for Co-Infection with Gastrointestinal Nematode in Anemic Meat Goats of Northeast Thailand

**DOI:** 10.3390/ani16030507

**Published:** 2026-02-05

**Authors:** Sarinya Rerkyusuke, Chariya Promphak, Pattiya Wongpattaraworakul, Pimchanok Taikitsayakun, Warisa Chuduang, Thakorn Thanaakkarasophon, Worakamol Chonsirikraisri, Julamanee Suriyapoom, Suthida Chanlun, Prapan Kaenjampa, Sawarin Lerk-u-suke, Peerapol Sukon, Patchara Phuektes

**Affiliations:** 1 Division of Livestock Medicine, Faculty of Veterinary Medicine, Khon Kaen University, Khon Kaen 40002, Thailand; sarinyare@kku.ac.th; 2 KKU Research Program, Khon Kaen University, Khon Kaen 40002, Thailand; julamanee2539@gmail.com (J.S.); sukonp@kku.ac.th (P.S.); 3 Faculty of Veterinary Medicine, Khon Kaen University, Khon Kaen 40002, Thailand; chariya.pr@kkumail.com (C.P.); pattiya.pw@kkumail.com (P.W.); orphic@kkumail.com (P.T.); warisa_ch@kkumail.com (W.C.); thakorn.th@kkumail.com (T.T.); worakamol.chon@kkumail.com (W.C.); 4 Laboratory Service and Laboratory Animal Unit, Faculty of Veterinary Medicine, Khon Kaen University, Khon Kaen 40002, Thailand; sutvir@kku.ac.th (S.C.); prapanka@kku.ac.th (P.K.); 5 Department of Geographic Information Science, School of Information and Communication Technology, University of Phayao, Phayao 56000, Thailand; sawarin.l@gmail.com; 6 Research Unit of Spatial Innovation Development, School of Information and Communication Technology, University of Phayao, Phayao 56000, Thailand; 7 Division of Anatomy, Faculty of Veterinary Medicine, Khon Kaen University, Khon Kaen 40002, Thailand; 8 Division of Pathobiology, Faculty of Veterinary Medicine, Khon Kaen University, Khon Kaen 40002, Thailand

**Keywords:** hemotropic mycoplasmosis, anemia, prevalence, risk factor, meat goat, northeast, Thailand

## Abstract

Hemotropic mycoplasmosis is an emerging vector-borne infection in goats caused by hemotropic mycoplasmas, which leads to anemia, poor growth, abortion, and reduced productivity. In Northeastern Thailand, smallholder meat goat systems expose animals to blood-feeding vectors and gastrointestinal parasites; however, epidemiological data on these issues are limited. This study examined the prevalence of hemotropic mycoplasmosis, its co-infection with gastrointestinal parasites, and the associated risk factors in anemic goats. PCR and sequence analyses confirmed the presence of *Mycoplasma ovis*, *Candidatus* Mycoplasma haematobovis and *Candidatus* Mycoplasma haematovis, with herd-level and individual-level prevalences of all hemotropic mycoplasmas (HMs) at 93.3% (14/15; 95% CI: 68.1–99.8%) and 59.8% (52/87; 95% CI: 48.7–70.1%), respectively. *Ca.* M. haematobovis was identified as the predominant species. Multivariable analysis indicated that age ≥ 1 year is a significant risk factor for HM infection (adjusted OR: 9.88; 95% CI: 1.73–56.48; *p =* 0.01). Co-infection between HM and gastrointestinal nematodes was found to be associated with age (*p* < 0.05). Effective management requires targeted selective treatment, post-treatment monitoring of infection status, vector control, and farmer education on zoonotic risks. These findings provide critical insights for designing evidence-based surveillance, control, and prevention strategies to improve goat health and productivity in smallholder tropical systems.

## Simple Summary

In Northeastern Thailand, meat goats are commonly raised in small-scale, free-ranging systems, which increase their exposure to blood-feeding vectors and gastrointestinal parasites. These infections can cause anemia, poor growth, and reduced productivity. Hemotropic mycoplasmosis, caused by *Mycoplasma ovis*, *Candidatus* Mycoplasma haematobovis and *Candidatus* Mycoplasma haematovis, is an emerging disease in goats, yet little is known about its prevalence in the region. This study investigated the occurrence and risk factors associated with hemotropic mycoplasmosis in meat goats, emphasizing the role of co-infection with gastrointestinal parasites and the potential zoonotic risk. The findings highlight the need for farmer education, targeted treatment, vector control, and integrated herd management to improve goat health and productivity.

## 1. Introduction

Goat production is becoming an increasingly important component of the livestock sector in many developing regions, contributing to household income, food security, and local economic stability [1]. In Thailand, goats are primarily raised for meat production, and the industry has expanded considerably over the past decade. At the national level, the goat population increased from 832,533 head in 2019 to 1,568,059 head in 2023, representing an overall increase of 88.35%. A similar trend was observed in the Northeastern region, where the number of goats rose from 96,489 to 338,779 head during the same period, corresponding to a 251.16% increase in population size. Concurrently, the number of registered goat producers grew substantially, from 3821 farmers in 2019 to 14,921 in 2023, reflecting a 290.51% increase and indicating broader engagement in goat production systems nationwide [2]. This growth has been driven by rising market demand, supportive agricultural policies from the government, and the suitability of goats for smallholder and resource-limited farming systems [3].

Goat production in Northeastern Thailand is primarily small-scale and community-based, with animals often grazed in semi-intensive or free-ranging systems. These practices often involve mixed grazing with other ruminant livestock on shared pastures, increasing animal contact and exposure to blood-feeding vectors [4,5]. The region’s tropical climate, characterized by high humidity and year-round warmth [4,6], supports the survival and reproduction of vectors, thereby elevating the risk of vector-borne disease transmission.

Parasitic infections, both internal and external, significantly compromise goat health and productivity. Hemoparasitic infections contribute to anemia, abortion, and poor growth performance, while gastrointestinal nematodes (GINs) can exacerbate anemia, suppress immune function, and reduce body condition, ultimately leading to decreased productivity [4,7,8]. In Thailand, hemoparasitic infections in goats include *Theileria* spp. [9,10], *Babesia* spp. [11], and *Anaplasma* spp. [9,11,12]. These vector-borne hemoparasites are primarily transmitted by ticks and other blood-feeding arthropods. Specifically, *Babesia* spp. are transmitted exclusively by ticks [13,14], while *Theileria* spp. and *Anaplasma* can also be transmitted mechanically by stable flies (*Stomoxys calcitrans*) [15,16,17,18]. Additionally, *Anaplasma* spp. may be transmitted iatrogenically through contaminated instruments [19].

Hemotropic mycoplasmas (HMs) are a group of vector-borne hemoparasites affecting livestock worldwide [20]. These cell wall-deficient, erythrocyte-associated bacteria infect a wide range of mammalian hosts, including goats [20]. Previously classified as *Haemobartonella* and *Eperythrozoon*, these organisms have been reassigned to the genus *Mycoplasma* based on molecular and phylogenetic analyses of 16S rRNA [21,22]. HM infections in goats have been reported worldwide, with *Mycoplasma ovis* identified as the predominant species across Europe [23,24], Africa [25], Asia [26,27,28,29], Australia [30], and the Americas [31,32,33]. Other HM species detected in goats include *Candidatus* Mycoplasma haemoovis in China, Hungary, and Thailand [23,27,34]; *Candidatus* Mycoplasma haemominutum in the Philippines [28]; and *Candidatus* Mycoplasma erythrocervae in Uganda [25]. In contrast, *Candidatus* Mycoplasma haematobovis has primarily been reported in cattle and buffalo [35,36].

The prevalence of HM infections varies significantly among geographic regions and production systems [25,26,27,28,29,31,32], indicating that environmental and management factors play a crucial role. Transmission is presumed to occur primarily through exposure to infected blood, particularly via hematophagous arthropods and iatrogenic procedures [20,30,37]. Additionally, vertical transmission has been confirmed in cattle and is suspected in small ruminants [20,37]. Although HM infections in goats are often subclinical, they can lead to adverse outcomes such as hemolytic anemia, impaired growth, reproductive losses, and reduced productivity, especially under stress or during co-infection with other parasites [27,38]. Notably, *M. ovis* has been identified as a zoonotic agent in humans [39,40].

Despite global reports, there is a lack of prior epidemiological data on HM infections in Northeastern Thailand, even though arthropod vectors are commonly observed in the region. Co-infection between HM and GIN may exacerbate clinical outcomes, including poor body condition and immunosuppression. Given the growing significance of meat goat production in Northeastern Thailand and the frequent occurrence of anemia in local herds [2,3,4], HM infection represents a potentially underrecognized health concern. Therefore, this study aims to determine the molecular prevalence of HM and identify associated risk factors for co-infection with GIN in anemic meat goats in Northeastern Thailand. The findings provide evidence to support targeted surveillance, prevention, and control strategies to enhance herd health and productivity in tropical smallholder production systems.

## 2. Materials and Methods

### 2.1. Ethical Statement

The study protocol underwent ethical evaluation and was authorized by the Institutional Animal Care and Use Committee (IACUC) (approval nos. IACUC-VM-KKU-710632.1.1/2.23, approved 22 May 2023; and IACUC-KKU(C)-21/67, approved 27 March 2024). Approval of research components involving human participants was granted by the Center for Ethics in Human Research (approval no. HE672102; approved 28 May 2024). All study activities adhered to institutional and national ethical requirements for research involving animals and humans.

To ensure confidentiality, all individual-level information was de-identified and reported only in aggregated form at the village level. Spatial representations were intentionally generalized to prevent the identification of specific households or participants.

### 2.2. Study Area and Design

These areas experience annual temperature variations ranging from 24.1 °C to 35.0 °C, with average rainfall between 0.0 mm and 12.7 mm. Relative humidity fluctuates from 49.6% to 95.6%, and the average atmospheric pressure is 1006.2 hPa, varying from a minimum of 1002.8 hPa to a maximum of 1013.7 hPa. Wind speeds range between 1.26 and 15.2 knots, while water evaporation is estimated at 3.0–7.9 mm per day [6]. This study was conducted from June to October 2023 during the monsoon season.

The study encompassed 15 meat goat herds located in Chaiyaphum and Khon Kaen provinces. In Chaiyaphum, herd sampling was carried out in Ban Thaen (4 herds), Kaset Sombun (2 herds), and Noen Sa-nga (2 herds). In Khon Kaen, herds were recruited from Ban Haet (1 herd), Khao Suan Kwang (2 herds), Nong Ruea (2 herds), and Si Chomphu (2 herds).

Clinical examination included anemia assessment using the FAMACHA scoring system, which grades conjunctival mucous membrane color on a five-point scale from 1 (non-anemic) to 5 (very severe anemia) [41]. Age was estimated by dentition, classifying goats as young (<1 year) when only deciduous teeth were present, and as adults (≥1 year, including both 1–4 years and >4 years) when permanent teeth were observed. Body condition scoring (BCS) was performed in accordance with Villaquiran et al. [42] using palpation of the lumbar and sternal areas, with scores ranging from 1 (obese) to 5 (emaciated).

### 2.3. Study Animals, Sampling Procedures and Data Collection

The estimated meat goat population in Chaiyaphum and Khon Kaen provinces is 63,015 animals [43]. The sample size was calculated based on an expected HM prevalence of 15%, as reported previously [34], with a 5% precision level, a design effect of 1.0, and a single-cluster assumption at an 80% confidence level. Under these parameters, the minimum required sample size was determined to be 84 goats, calculated using Epi Info™ for Windows (version 7.2.5.0), with the formula shown below.

Sample size (n) = [*DEFF* × *N*p(1 − p)]/[(*d*^2^/Z^2^_1−α/2_ × (*N* − 1) + p × (1 − p)]

Population size (for finite population correction factor or fpc) (*N*): 63,015
Hypothesized % frequency of an outcome factor in the population (p): 15% ±5
Confidence limits as % of 100 (absolute ± %) (*d*): 5%
Design effect (for cluster surveys-*DEFF*): 1.0
Confidence Level (%): 80%
Sample size (n): 84

The study was conducted in smallholder meat goat herds managed under communal production systems. Herd enrollment utilized both convenience-based and random approaches, with participation depending on farmers’ consent. Fifteen smallholder meat goat herds were enrolled, with herd sizes ranging from 11 to 95 goats (mean: 42.2; SD: 27.9; 95% CI: 26.7–57.7). None of the enrolled herds had received anthelmintic or antibiotic treatments for at least one month prior to sampling.

A total of 633 goats from 15 enrolled farms were clinically examined. Eligible goats were those with clinical anemia (FAMACHA score > 3) and older than 2.5 months, and included both sexes across multiple age groups. A total of 87 anemic goats from 15 herds were included in the study. For each animal, individual records included identification, age, and sex, as well as clinical observations such as FAMACHA score, body condition score (BCS), presence of submandibular edema, and hydration status. Blood and fecal samples were collected from each goat during a single visit. Blood samples were obtained from all goats; fecal samples were collected only from individuals that had feces at the time of sampling and thus were not obtained from some animals.

Blood sampling was performed aseptically by jugular venipuncture, with a total volume of 6 mL collected from each goat. The sample was divided into a plain tube (4 mL) and an EDTA tube (2 mL). Immediately after collection, the samples were kept on ice and delivered to the laboratory at the Faculty of Veterinary Medicine, Khon Kaen University, within 6 h. Following centrifugation at 2500 rpm for 10 min, serum was separated and stored at −20 °C until analysis.

Fresh fecal material (at least 10 g) was collected directly from the rectum of 76 goats (with no feces obtained from 11 goats) and placed in sealed plastic bags. The fecal samples were maintained at 4 °C during transport and storage prior to laboratory processing at the same institution.

### 2.4. Laboratory Analysis

#### 2.4.1. Blood and Serum Analysis

Packed cell volume (PCV, %) was measured from EDTA-anticoagulated blood using the microhematocrit method after centrifugation at 3000 rpm for 15 min. PCV was expressed as the percentage of erythrocytes relative to the total blood volume. Following the PCV assessment, approximately 1.5 mL of EDTA blood was stored at −20 °C for molecular analyses. Total serum protein concentration (g/dL) was determined using a handheld refractometer (Erma, Model D, Tokyo, Japan).

#### 2.4.2. Fecal Analysis

Fecal egg counts were performed using the method described by Brummaier et al. [44], and helminth eggs were identified based on morphological characteristics, following the standard diagnostic criteria outlined by Taylor et al. [45].

#### 2.4.3. Detection of Hemotropic Mycoplasmas by Conventional PCR

##### DNA Extraction

DNA was extracted from EDTA-treated blood samples using the QIAamp^®^ DNA Mini Kit (Qiagen, Hilden, Germany) according to the manufacturer’s guidelines. Briefly, 200 µL of blood was processed through enzymatic lysis and column-based purification, after which DNA was eluted in the provided buffer and stored at −20 °C until molecular analyses.

##### PCR Amplification and Sequencing

Detection of *Mycoplasma* spp. was performed using conventional PCR targeting the 16S rRNA gene with the primer pair MycoHBT-F (5′-ATACGGCCCATATTCCTACG-3′) and MycoHBT-R (5′-TGCTCCACCACTTGTTCA-3′), as previously reported by Criado-Fornelio et al. [46]. Amplification reactions were carried out in a Bio-Rad^®^ thermal cycler (Bio-Rad Laboratories, Inc., Hercules, CA, USA) in a final volume of 25 µL, containing 2 µL of template DNA, primers at a final concentration of 0.4 µM each, and 12.5 µL of 2 × GoTaq^®^ Green Master Mix (Promega, Madison, WI, USA). The cycling protocol consisted of an initial denaturation at 95 °C for 5 min, followed by 35 cycles of denaturation at 95 °C for 30 s, annealing at 55 °C for 30 s, and extension at 72 °C for 1 min, with a final extension step at 72 °C for 7 min.

Amplified products were resolved on a 1.5% (*w*/*v*) agarose gel prepared in 1× TBE buffer and visualized using RedSafe™ nucleic acid stain (INtRON Biotechnology, Seongnam, Republic of Korea). DNA extracted from *Mycoplasma* spp. served as a positive control, whereas nuclease-free water was included as a negative control. Fragment sizes were estimated using a 100 bp Plus DNA ladder (Vivantis, Subang Jaya, Malaysia). Selected amplicons of the expected size were randomly chosen for purification and sequencing; a total of 19 PCR products derived from 11 herds were processed. Purification was conducted using the GF-1 AmbiClean Kit (Vivantis, Subang Jaya, Malaysia), and sequencing was performed by ATGC Co., Ltd., (Pathum Thani, Thailand).

#### 2.4.4. DNA Sequence and Phylogenetic Analysis of Hemotropic Mycoplasmas

The individual nucleotide sequences and their corresponding chromatograms from each sample were assessed using Chromas software version 2.6.6 (https://technelysium.com.au/wp/chromas/, accessed on 2 April 2025). Following manual adjustments, these nucleotide sequences were aligned with the NCBI database using the nucleotide BLAST tool version 2.17.0 (https://blast.ncbi.nlm.nih.gov/Blast.cgi/, accessed on 2 April 2025) to determine the species of the organisms.

The partial sequences of the 16S rRNA gene for various hemotropic mycoplasmas, including *M. haemofelis* (PP494718.1), *M. haemocanis* (KU765208.1), *Candidatus* Mycoplasma haematobovis (PQ433656.1, EU367965.1, AB740010.1), *M. wenyonii* (OP394159.1), *M. ovis* (AF338268.1, EU165509.1, KU983741.1, CP006935.1), *Candidatus* Mycoplasma haematovis (KU983747.1, KU983749.1, JF931131.1), and *Candidatus* Mycoplasma haematoparvum (PQ149020.1), were retrieved from the GenBank^®^ database and utilized for constructing the phylogenetic tree. Nucleotide sequence alignment was conducted using the MUSCLE algorithm within MEGA software version 12 (https://www.megasoftware.net/, accessed on 2 April 2025). Phylogenetic analyses were performed using the Neighbor-Joining method with the Maximum Composite Likelihood model for nucleotide substitution, and branch support was assessed by bootstrap analysis with 1000 replicates.

### 2.5. Questionnaires

Fifteen herd owners were interviewed between May and July 2024 to assess risk factors associated with HM infections within their herds. The risk factors were analyzed at two levels: herd level and individual level.

Herd-level risk factors included farmers’ knowledge and awareness of caprine hemoparasitic diseases, clinical signs in animals and humans suggestive of hemotropic mycoplasmosis, herd management and animal health care practices, the presence of other livestock species on the farm, observation of potential disease vectors and evidence of bites or blood-feeding on animals, the presence of insect breeding sites on the premises, grazing patterns and pasture usage, animal movement and quarantine practices, history of sharp instrument use on the farm, breeding history and mating methods, barn sanitation practices, and vector control measures implemented on the farm. Individual-level risk factors included gender, age, BCS, and FAMACHA score.

### 2.6. Data Analysis

Data management was performed in Microsoft Excel, and statistical analyses were conducted using IBM SPSS Statistics (version 29.0; IBM Corp., Armonk, NY, USA) and MedCalc^®^ (version 22.021; MedCalc Software Ltd., Ostend, Belgium, 2024). Data distribution was evaluated using the Shapiro–Wilk test. Summary statistics, including measures of central tendency and dispersion (mean, median, standard deviation [SD], and interquartile range [Q1–Q3]), as well as 95% confidence intervals (CIs), were calculated as appropriate.

Associations between HM infections and potential herd-level or animal-level factors were initially screened using univariate logistic regression analyses, with statistical significance defined as *p* < 0.05. Odds ratios (ORs) and corresponding 95% CIs were used to quantify associations. To prevent potential overlooking of critical confounders or variables that only achieve significance when adjusted for other covariates, all biologically relevant variables (Gender, Age, BCS, FAMACHA score, PCV, Total protein, and Nematode) were included in the multivariable logistic regression model. Then, the adjusted ORs with 95% CIs are reported.

Spearman’s rank correlation was performed to evaluate relationships among age, BCS, FAMACHA score, PCV, total protein, nematode parasite burden (EPG), and HM infections, with statistical significance set at *p* < 0.05. As most variables were not normally distributed (Shapiro–Wilk test, *p* < 0.05), nonparametric analysis was used.

Spatial analysis and visualization were performed using Geographic Information Systems (GIS) in QGIS (version 3.36.0).

## 3. Results

### 3.1. Descriptive Summary of Anemia in Goats in Relation to Age, Body Condition Score (BCS), FAMACHA Score, Packed Cell Volume (PCV), and Total Protein

Among the 633 goats examined across 15 farms, 87 were classified as anemic. At the herd level, the median number of anemic goats was 4 per farm (interquartile range [IQR]: 2–10), with counts ranging from 1 to 19 animals. The distribution of anemic goats according to age group, BCS, and FAMACHA score category is presented in Table 1. Age-stratified values of PCV and total protein are summarized in Table 2.

### 3.2. Prevalence of Gastrointestinal Nematodes in Anemic Goats

Fecal samples were obtained from 76 of the 87 anemic goats across 15 farms. GIN infection was detected on all farms (100%; (15/15)). At the individual level, 98.6% of goats (75/76) tested positive for nematode eggs, with only one sample showing a negative result. The median fecal egg count was 193 EPG (range: 0–4158; IQR (Q1–Q3): 63.5–570.75).

The number of anemic goats by age group and nematode infection is presented in Table 3, while age-stratified distributions of fecal egg counts from GIN infections are shown in Table 4.

### 3.3. Prevalence of Hemotropic Mycoplasma (HM) Infections in Meat Goat Herds

**Figure 1:**
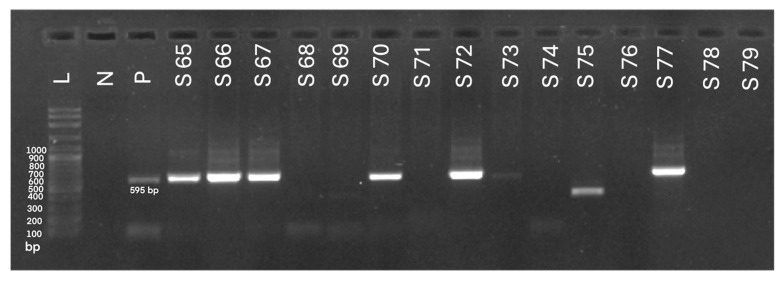
Agarose gel electrophoresis (1.5%) demonstrating PCR amplification of the 16S rRNA gene (595 bp) of hemotropic mycoplasmas. L: DNA ladder; N: negative control; P: positive control (*Mycoplasma* spp.); S65–67, S70, S72–73, S77: PCR amplicons from infected animals; S68–69, S71, S74–76, S78–79: samples from uninfected animals.

The herd-level molecular prevalence of HM was 93.3% (14/15). At the individual level, 59.8% (52/87) of goats tested PCR-positive for hemotropic mycoplasma DNA in blood samples. Representative PCR-positive amplicons are presented in Figure 1. The prevalence within herds ranged from 0% to 100% (95% confidence interval [CI]: 43.7–81.2), as summarized in Table 5.

### 3.4. Association Between Parasitic Infections, Age, Clinical Signs, Packed Cell Volume (PCV) and Total Protein Results

Of the 76 goats from which both blood and fecal samples were collected, one tested negative for GIN, and another tested negative for HM. Spearman’s rank correlation was performed to determine associations among age, clinical signs, hematological, biochemical, and parasitological parameters in the remaining 74 animals, revealing significant associations. Age was positively correlated with BCS (r = 0.23, *p* < 0.05), PCV (r = 0.24, *p* < 0.05), total protein (r = 0.38, *p* < 0.001), and the presence of nematode infection (r = 0.31, *p* < 0.01). BCS was negatively associated with PCV (r = −0.23, *p* < 0.05). The FAMACHA score exhibited strong negative correlations with PCV (r = −0.53, *p* < 0.001) and total protein (r = −0.31, *p* < 0.01). PCV was positively associated with total protein (r = 0.64, *p* < 0.001) and negatively correlated with nematode EPG (r = −0.49, *p* < 0.001), while total protein was also negatively correlated with nematode EPG (r = −0.54, *p* < 0.001). Co-infection with nematodes and HM showed a weak positive correlation with age (r = 0.28, *p* < 0.05), whereas HM infection alone was not significantly associated with any measured parameter.

Overall, these findings indicate that anemia severity and protein status are closely linked with GIN burden, whereas HM infection appears largely independent of clinical signs, as well as hematological and biochemical variables in this cohort. Correlations between age, BCS, FAMACHA score, PCV, total protein, and parasite infections are summarized in Table 6.

### 3.5. Spatial Distribution of Hemotropic Mycoplasma (HM) Infections in Meat Goat Herds

**Figure 2:**
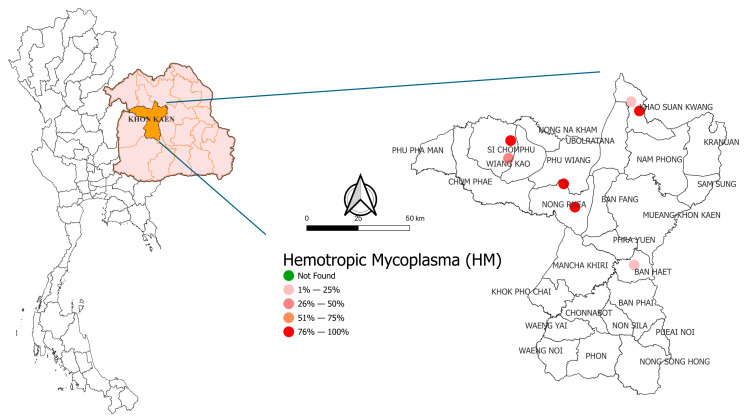
Molecular prevalence and distribution of hemotropic mycoplasma (HM) infections in meat Goats in Khon Kaen province.

**Figure 3:**
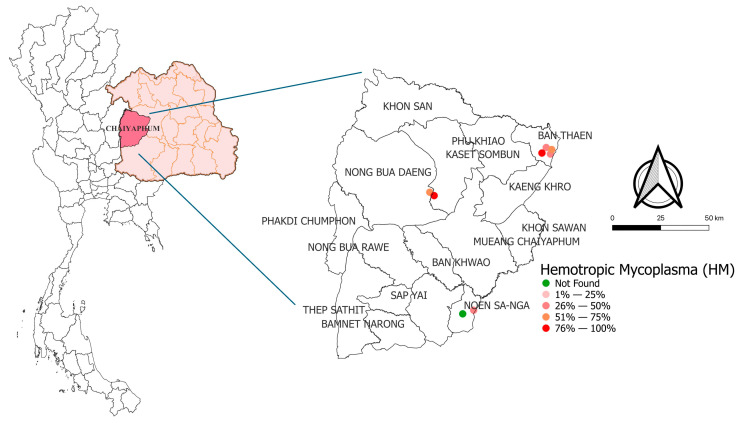
Molecular prevalence and distribution of hemotropic mycoplasma (HM) infections in meat goats in Chaiyaphum province.

HMs were detected in both provinces surveyed. In Khon Kaen Province, all four districts and all seven farms examined tested positive for the infection. In Chaiyaphum Province, HMs were detected in all districts, except for one farm in Noen Sa-nga District, which tested negative. The molecular prevalence and distribution of HM infections in meat goats in Khon Kaen and Chaiyaphum are presented in Figure 2 and Figure 3, respectively.

### 3.6. Sequence and Phylogenetic Analysis of Hemotropic Mycoplasmas (HM) in Meat Goat Herds

Sequence analyses were performed on 19 PCR products selected randomly to represent each of the 11 farms. Samples from 3 other positive farms were not sequenced due to insufficient DNA concentrations. Based on the BLAST sequence analysis, *Ca.* M. haematobovis and *M. ovis* were detected in both provinces, with *Ca.* M. haematobovis being the most prevalent species identified, present in 8 of the 11 farms investigated, followed by *M. ovis*, which was found in 3 farms. *Ca.* M. haematovis was detected in 2 farms exclusively within Chaiyaphum Province. Among the 11 farms analyzed, mixed-species infections were observed in 2 farms, while single-species infections were found in 9 farms. The sequence analysis of hemotropic mycoplasmas in meat goats, as indicated by the BLAST results from PCR-positive blood samples, is summarized in Table 7.

**Figure 4:**
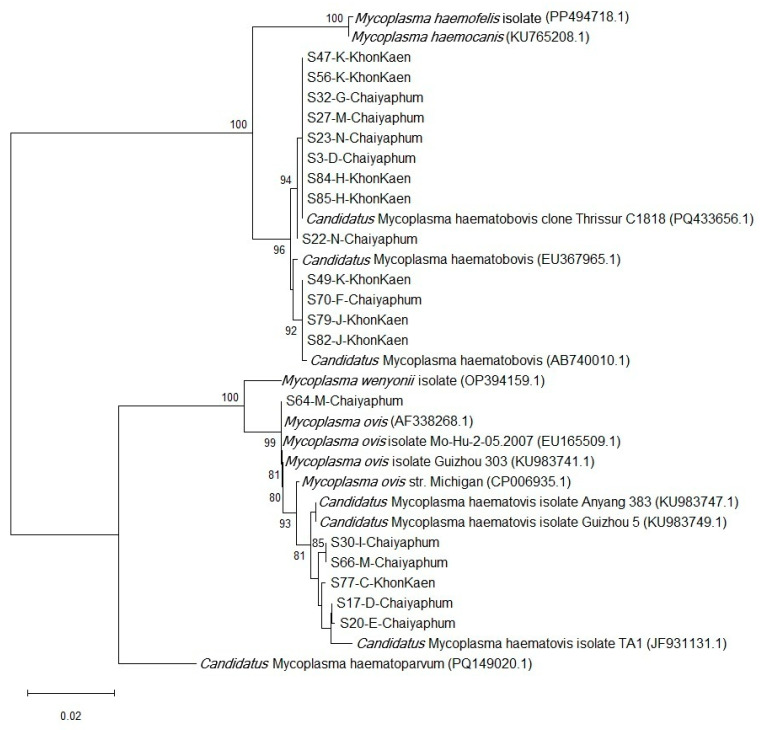
The phylogenetic tree of hemotropic mycoplasmas (HMs) based on the partial nucleotide sequences of the 16S rRNA gene using the Neighbor-Joining method, with the Maximum Composite Likelihood model for nucleotide substitution. Numbers at the nodes indicate the percentage occurrences of clades, derived from 1000 bootstrap replications of the dataset.

Phylogenetic analysis of the partial 16S rRNA gene nucleotide sequences revealed that the isolates in this study cluster were within the same clade as *Ca.* M. haematobovis, *Ca.* M. haematovis, and *M. ovis*, but not with other hemotropic mycoplasmas. Isolates from both provinces are grouped within the same clades, indicating a common origin or recent transmission. Notably, three isolates identified as *M. ovis* strain Michigan by BLAST analysis were classified as *Ca.* M. haematovis. Phylogenic tree of HM is presented in Figure 4.

### 3.7. Risk Factors Associated with Hemotropic Mycoplasma (HM) Infection in Meat Goat Herds

Evaluation of herd-level risk factors for HM infection indicated that none of the assessed variables were significantly associated with infection (*p* > 0.05). The results of the univariate analysis at the herd level are summarized in Table 8.

At the individual level, goats older than one year had significantly higher odds of HM infection compared to younger animals (OR: 7.09; 95% CI: 2.07–24.23; *p = 0.002*). Similarly, goats with a poor BCS (BCS ≥ 3.5) exhibited significantly higher odds of infection than those with a good BCS (BCS ≤ 3) (OR: 23.14; 95% CI: 1.25–425.38; *p = 0.035*). No significant associations were observed for gender, FAMACHA score, PCV, total protein concentration, or the severity of GIN infection (*p* > 0.05). The univariate analysis of individual-level risk factors for HM is presented in Table 9. Subsequent multivariable analysis identified age as a significant risk factor associated with HM in meat goat herds (*p* < 0.05); results for other biologically relevant factors are also reported in Table 10.

## 4. Discussion

Anemia observed in this study reflects a complex interaction among GIN infection, host age, nutritional status, and physiological stress, with HM acting as a secondary modifier rather than a primary cause. Low BCS was consistently associated with elevated FAMACHA scores, reduced PCV, and decreased total protein concentrations, indicating chronic undernutrition and sustained blood and protein loss, which are hallmark consequences of GIN infection, particularly Strongyle-type nematodes [4,49,50]. These parasites induce anemia through hematophagy, intestinal mucosal damage, and impaired nutrient absorption, leading to prolonged reductions in erythrocyte mass and circulating proteins [51].

The age-related patterns observed in this study indicate that anemia and infection risk in goats are driven by cumulative physiological and environmental stress rather than age per se. Young goats (<1 year) exhibited the most severe anemia in parallel with poor body condition and high fecal egg counts, reflecting the combined pressures of rapid growth, elevated nutritional requirements, and immature immune defenses that limit effective control of gastrointestinal nematodes [4,45,49]. Conversely, older goats (>4 years) maintained persistently reduced PCV and total protein concentrations despite lower egg outputs, supporting a pattern of progressive hematological depletion associated with repeated parasitic exposure and chronic nutritional insufficiency rather than recent infection events [50,52].

The age of the animals was statistically associated with HM infection, while neither BCS nor PCV independently predicted HM positivity, indicating that HM persistence is not directly driven by overt anemia or poor nutritional status. Age likely serves as a proxy for prolonged exposure to arthropod vectors and contaminated environments, thereby increasing cumulative infection pressure over time [28,53,54]. The inconsistent age–infection relationships reported in previous studies [26,27,33,34] further reinforce that HM epidemiology is shaped by interacting factors—including co-infections, immune resilience, husbandry practices, and environmental conditions rather than age in isolation.

A high prevalence of HM was observed at both the herd and individual levels in this study conducted in northeastern Thailand. In contrast, previous investigations of goats from northern, eastern, western, central, and southern Thailand reported a substantially lower prevalence of 15.5% [34]. The markedly higher detection rate observed in this study is likely attributable, at least in part, to regional differences in epidemiological conditions that may influence the prevalence of HM infections. HM infection exhibited only weak independent associations with hematological (PCV) and biochemical (total protein) indices, consistent with its predominantly subclinical, low-bacteremia infections [27,31]. In contrast, the significant age-related pattern of HM–GIN coinfection provides strong evidence that sustained parasitic pressure and nutritional stress facilitate HM persistence and exacerbate anemia severity. Collectively, these findings support a model in which HM functions primarily as a secondary stressor that worsens anemia under concurrent gastrointestinal parasitism and compromised host condition, rather than acting as a primary etiological agent [8,38,55].

Molecular analysis identified the presence of *M. ovis* and *Ca.* M. haematobovis in both provinces. However, *Ca.* M. haematovis was detected solely on two farms in Chaiyaphum province. The most predominant species identified was *Ca.* M. haematobovis, found in 72.7% (8/11) of the farms where samples were sequenced. The high prevalence of *Ca.* M. haematobovis observed here is consistent with molecular prevalence rates reported in China, where it was detected in 45.2% and 58.2% of animals in 2018 and 2022, respectively [56,57]. These findings collectively suggest that *Ca.* M. haematobovis may have a central role in the epidemiology of HM within the region’s small ruminant populations. First identified in Japanese cattle between 2005 and 2007 [58], *Ca.* M. haematobovis has since been reported in cattle across a wide range of global locations, including China, Germany, England, Brazil, Mexico, and Iraq [14,59,60,61,62,63]. In addition to cattle, *Ca.* M. haematobovis infections have been documented in various hosts, including water buffalo [59,64], wild cervids [53], and other wild ruminants [35]. Infections have also been identified in goats, sheep, and even dogs [27,56,57,65]. The expanding host range and widespread geographic distribution of *Ca.* M. haematobovis highlights its importance as an emerging hemotropic pathogen with potential implications for animal health, livestock productivity, and cross-species transmission. The high prevalence documented in the goat population in this study underscores the critical need for enhanced surveillance, vector control, and improved farm management practices to mitigate the impact of this pathogen on small ruminants.

Interestingly, the isolates identified as *M. ovis* strain Michigan by BLAST analysis were classified as *Ca.* M. haematovis in the phylogenetic analysis, indicating a close genetic relationship between these two species. Our findings are consistent with previous studies [27,34], suggesting that further analysis of nucleotide sequences of additional gene markers is necessary for more accurate identification. Similar to other regions in Thailand that reported prevalence rates of *M. ovis* in healthy goats ranging from 1.7% to 42.5% [34], our study found the pathogen in meat goats from the northeastern region. Higher prevalence rates of *M. ovis* have been documented internationally, with reports from China, Japan, and Hungary showing rates of 45%, 50%, and 51.5%, respectively [27,53,58]. This variation suggests that the prevalence of *M. ovis* can differ significantly by region. *M. ovis* infections have also been identified in various hosts, including sheep, deer, reindeer, and humans [24,27,40,66,67,68,69,70]. *Ca.* M. haematovis, detected in our study, was first identified in sheep in Hungary [53]. Subsequent studies have documented its presence in small ruminants in Japan and China [27,71]. Therefore, further investigation is essential to thoroughly examine the prevalence of these two zoonotic pathogens in goats and other susceptible hosts in northeastern Thailand.

Although herd-level risk factors for HM infections were not significantly different between infected and non-infected herds in this study, most infected herds had other livestock or companion animals present, as well as vectors on the farm. This suggests potential for cross-transmission of HM among livestock and companion animals in this endemic area. Natural transmission is strongly influenced by blood-sucking vectors such as mosquitoes, stable flies, and ticks [25,28]. All infected farms in this study had mosquitoes, with nearby water sources promoting breeding. Stable flies were present on 46.7% of farms, corresponding to a 40% infection rate in goats, as these flies prefer warm, moist environments with decomposing organic matter [72,73]. Stomoxys flies, which are capable of long-distance flights [74], were found on 33.3% of infected farms. Ticks were associated with the lowest infection rate (20%), likely due to their host-specific life cycle and limited dispersal compared to flying vectors [75].

In terms of vector-borne transmission, Arendt et al. [20] reviewed hemotropic mycoplasma DNA in mosquitoes, horn flies, stable flies, tabanid flies, ticks, and chewing lice. *M. ovis* has been identified in mosquitoes (*Aedes camptorhynchus*, *Culex annulirostris*), stable flies (*Stomoxys* spp.), chewing lice (*Bovicola ovis*), ticks (*Rhipicephalus microplus*) and, more recently, in fleas (*Ctenocephalides orientis*) [34]. Vectors of *Ca.* M. haematobovis include horn flies (*Haematobia irritans*), stable flies (*Stomoxys calcitrans*), tabanid flies (*Tabanus bovinus*, *Tabanus bromius*), and ticks (*Rhipicephalus microplus*, *Haemaphysalis longicornis*) [20]. Additionally, *Ca.* M. haematovis has also been detected in *Hyalomma dromedarii* ticks [76] and *Ctenocephalides orientis* fleas [34]. Proximity to neighboring livestock, particularly beef cattle farms within 500 m of goat herds, was associated with an increased risk of infection. Besides natural transmission, iatrogenic factors such as the use of shared needles or contaminated equipment may also contribute to pathogen dissemination within herds [21,32]. These findings underscore the multifactorial nature of HM transmission, involving vector ecology, farm management practices, and interspecies proximity, and highlight the urgent need for integrated surveillance and control measures in endemic regions.

This study revealed a substantial knowledge gap among farmers, with 93% lacking prior awareness of the disease and only one reporting a suspected human case. Given that *M. ovis* have been associated with human infections [40,66,70,77], educating farmers on zoonotic prevention is essential. Surveillance should also extend to other livestock, companion animals, and humans to assess pathogen dissemination. These data can inform evidence-based strategies for diagnosis, control, and prevention, thereby strengthening disease management and mitigating further transmission in endemic areas.

A key limitation of this study is that only anemic goats were examined; consequently, clinically healthy animals that may serve as subclinical carriers were not included. Other limitations include the small sample size (only 15 herds examined) and incomplete fecal sampling, as specimens were unavailable from some animals during the visit. These issues may limit the generalizability of the findings and warrant caution when interpreting associations with potential risk factors, which may have been affected by missing data. Additionally, other livestock species, companion animals, wildlife, and potential arthropod vectors were not investigated, despite their possible roles in local transmission and pathogen persistence. Future studies should incorporate systematic surveys of clinically healthy animals, along with multi-host and vector assessments, utilizing large sample sizes to better elucidate transmission dynamics within herds and across the production environment. Effective management of GIN and HM infections should integrate targeted anthelmintic treatment guided by FAMACHA scoring, nutritional supplementation, and regular hematological monitoring to reduce anemia and improve overall goat health and productivity.

## 5. Conclusions

A considerable prevalence of HM was noted at both the herd and individual levels among smallholder meat goat farms in Northeastern Thailand. *Ca.* M. haematobovis was the predominant species, highlighting its epidemiological significance, while the detection of *M. ovis* and *Ca.* M. haematovis suggests potential zoonotic implications. Anemia in goats was primarily associated with GIN infection and cumulative physiological stress, whereas HM infection was predominantly subclinical. Age-related trends reflected cumulative exposure rather than age itself. These findings support age-focused surveillance, targeted selective treatment, integrated vector control, and farmer education as key strategies to mitigate the impact of HM in endemic areas.

## Figures and Tables

**Table 1:** Distribution of anemic animals according to age, body condition score (BCS), and FAMACHA score.

Age (yr)	Total No. (*N*)	BCS (3–5)					FAMACHA Score (3–5)				
		3	3.5	4	4.5	5	3	3.5	4	4.5	5
<1	17	5	4	4	2	2	3	1	5	3	5
1	11	1	1	4	1	4		1	2		8
2	7			3	2	2			1	1	5
3	12		1	3	5	3	1		5	2	4
4	18		2	2	3	11	2	2	6	2	6
>4	22		5	6	4	7	2	5	6	2	7
Total	87	6	13	22	17	29	8	9	25	10	35
	(%)	6.90	14.94	25.29	19.54	33.33	9.20	10.34	28.74	11.49	40.23

*N* = number of animals.

**Table 2:** Packed cell volume (PCV) and total protein distributions according to age group.

Age (yr)	Total No. (*N*)	PCV (%)		Total Protein (g/dL)	
		Median	IQR (Q1–Q3)	Mean + SD	95% CI
<1	17	12.5	9.0–19.5	5.1 + 1.0	4.6–5.7
1	11	9.0	8.0–13.0	5.0 + 0.6	4.5–5.4
2	7	10.0	9.0–15.0	5.2 + 0.8	4.3–6.0
3	12	13.0	10.0–16.0	5.8 + 1.0	5.1–6.5
4	18	13.0	10.7–19.0	6.1 + 0.9	5.6–6.5
>4	22	15.5	12.0–20.5	6.0 + 1.0	5.6–6.5

*N* = number of animals; IQR = Interquartile Range; PCV = Packed cell volume (normal range = 22.0–38.0) [47]; Total protein (normal range = 6.2–7.9 g/dL) [48]; The PCV data did not follow a normal distribution, while the total protein data adhered to a normal distribution.

**Table 3:** Number of anemic goats by age group and gastrointestinal nematode (GIN) infection.

Age (yr)	Total No. (*N*)	No. of Fecal Samples * (*N*)	Nematode		
			Strongyle	*Strongyloides* spp.	*Trichuris* spp.
<1	17	16	16	1	3
1	11	10	10	1	1
2	7	7	7	1	1
3	12	9	9	2	3
4	18	15	15	1	6
>4	22	19 **	18		6
Total	87	76	75	6	20
(%) ***			86.20	6.90	22.99

*N* = number of animals or fecal samples; * Samples are missing from some animals. ** Only one sample tested negative for gastrointestinal parasites; *** Percentages were calculated by dividing the total number of positive samples by the total number of animals (87).

**Table 4:** Fecal egg counts of gastrointestinal nematode (GIN) infections stratified by age.

Age (yr)	Strongyle * (EPG)				*Strongyloides* spp. * (EPG)				*Trichuris* spp. * (EPG)			
	*N*	Median	Min–Max	IQR (Q1–Q3)	*N*	Median	Min–Max	IQR (Q1–Q3)	*N*	Median	Min–Max	IQR (Q1–Q3)
<1	16	163	15–4049	76.5–936.7	1	2	2	-	3	5	3–10	3–10.0
1	10	363	11–1141	144.5–592.2	1	638	638	-	1	25	25	-
2	7	160	54–1199	54.0–199.0	1	7	7	-	1	17	17	-
3	9	268	19–875	45.5–472.0	2	32	27–37	-	3	31	10–45	10–45.0
4	15	251	8–1709	77.0–826.0	1	2	2	-	6	17	2–223	11.7–88.0
>4	18	180	3–4158	11.0–446.2	0	-	-	-	6	31	7–115	7–65.5

*N* = number of animals; EPG = Egg count per gram; IQR = Interquartile Range; * All data did not follow a normal distribution.

**Table 5:** Molecular prevalence of hemotropic mycoplasmas (HM) in 15 meat goat herds, Northeast Thailand.

No.	ID Farm	Molecular Prevalence % (Numbers of PCR-Positive/Total Samples)
1	A	0.0 (0/1)
2	B	10.0 (1/10)
3	C	20.0 (1/5)
4	D	42.1 (8/19)
5	E	50.0 (1/2)
6	F	50.0 (2/4)
7	G	50.0 (2/4)
8	H	75.0 (3/4)
9	I	75.0 (3/4)
10	J	80.0 (4/5)
11	K	84.6 (11/13)
12	L	100.0 (10/10)
13	M	100.0 (1/1)
14	N	100.0 (4/4)
15	O	100.0 (1/1)
Total		59.8 (52/87)

**Table 6:** Spearman’s correlations among age, body condition score (BCS), FAMACHA score, packed cell volume (PCV), total protein, and parasite infections (n = 74).

Parameter	Age		BCS		FAMACHA Score		PCV		Total Protein	
	r=	*p*-Value	r=	*p*-Value	r=	*p*-Value	r=	*p*-Value	r=	*p*-Value
*Clinical sign*										
BCS	0.23	*	-		0.22	NS	−0.23	*	0.04	NS
FAMACHA score	−0.09	NS	0.22	NS	-		−0.53	***	−0.31	**
*Hematology*										
PCV (%)	0.24	*	−0.23	*	−0.53	***	-		0.64	***
*Biochemistry*										
Total Protein (g/dL)	0.38	***	0.04	NS	−0.31	**	0.64	***	-	
*Parasite infection*										
Nematode (EPG)	−0.12	NS	0.001	NS	0.21	NS	−0.49	***	−0.54	***
Nematode infection	0.31	**	0.20	NS	0.06	NS	0.12	NS	0.21	NS
HM infection	−0.15	NS	−0.14	NS	−0.12	NS	−0.10	NS	−0.06	NS
Co-infection; nematode and HM	0.28	*	0.16	NS	0.03	NS	0.10	NS	0.19	NS

* = *p* < 0.05; ** = *p* < 0.01; *** = *p* < 0.001; NS = not significant.

**Table 7:** BLAST sequence analysis of PCR-positive blood samples from goats infected with hemotropic mycoplasmas (HMs).

No.	Sample Number	ID Farm	Location (Province)	Identified Organisms by BLAST Closet Sequence	Length	Percent Identity (%)	Accession Number
1	S77 *	C	Khon Kaen	*M. ovis* str. Michigan *	543	98.9	CP006935.1
2	S3	D	Chaiyaphum	*Ca.* M. haematobovis	522	100	PQ433656.1
3	S17			*Ca.* M. haematovis	483	99.7	KU983747
4	S20	E	Chaiyaphum	*Ca.* M. haematovis	502	99.6	KU983747.1
5	S70	F	Chaiyaphum	*Ca.* M. haematobovis	522	99.81	AB740010.0
6	S32	G	Chaiyaphum	*Ca.* M. haematobovis	522	100	PQ433656.1
7	S84	H	Khon Kaen	*Ca.* M. haematobovis	522	100	PQ433656.1
8	S85			*Ca.* M. haematobovis	522	100	PQ433656.1
9	S30 *	I	Chaiyaphum	*M. ovis* str. Michigan *	543	98.9	CP006935.1
10	S79	J	Khon Kaen	*Ca.* M. haematobovis	522	99.81	AB740010.0
11	S82			*Ca.* M. haematobovis	522	99.81	AB740010.0
12	S47	K	Khon Kaen	*Ca.* M. haematobovis	522	100	PQ433656.1
13	S49			*Ca.* M. haematobovis	522	99.81	AB740010.0
14	S56			*Ca.* M. haematobovis	522	100	PQ433656.1
15	S27	M	Chaiyaphum	*Ca.* M. haematobovis	522	100	PQ433656.1
16	S64			*M. ovis*	543	100	AF338268.1
17	S66 *			*M. ovis* str. Michigan *	543	98.9	CP006935.1
18	S22	N	Chaiyaphum	*Ca.* M. haematobovis	522	99.81	PQ433656.1
19	S23			*Ca.* M. haematobovis	522	100	PQ433656.1

* Isolate was identified as *Ca*. M. haematovis in the phylogenetic tree.

**Table 8:** Univariate analysis of herd-level risk factors for hemotropic mycoplasma (HM) infections in meat goat herds in Northeastern Thailand.

Risk Factor	Category	Total (%)	Prev. (%)	OR	95% CI	*p*-Value
Knowledge of blood parasites	Not know	14 (93.3)	13/15 (86.7)	3.00	0.08–111.78	0.551
	Know	1 (6.7)	1/15 (6.7)			
Symptoms of animals susceptible to HM						
Anemia signs	Presence	8 (53.3)	8/15 (53.3)	3.92	0.13–112.90	0.425
	Absence	7 (46.7)	6/15 (40.0)			
Jaundice signs	Presence	2 (13.3)	2/15 (13.3)	0.60	0.01–19.41	0.773
	Absence	13 (86.7)	12/15 (80.0)			
Abortion	Presence	11 (73.3)	11/15 (73.3)	9.85	0.32–300.42	0.189
	Absence	4 (26.7)	3/15 (20.0)			
Weak-born kids	Presence	9 (60.0)	9/15 (60.0)	5.18	0.17–150.46	0.338
	Absence	6 (40)	5/15 (33.3)			
Lymph node enlargement	Presence	1 (6.7)	1/15 (6.7)	0.33	0.008–12.42	0.551
	Absence	14 (93.3)	13/15 (86.7)			
Red urine	Presence	0 (0.0)	0/15 (0.0)	0.10	0.001–7.36	0.297
	Absence	15 (100)	14/15 (93.3)			
Dead from anemia	Presence	2 (13.3)	2/15 (13.3)	0.60	0.01–19.41	0.773
	Absence	13 (86.7)	12/15 (80.0)			
Symptoms of human susceptibility to HM						
For example, acute fever or anemia or muscle pain or weakness or diarrhea or anorexia	Presence	1 (6.7)	1/15 (6.7)	0.33	0.008–12.42	0.551
	Absence	14 (93.3)	13/15 (86.7)			
How to treat in human	Get drug by yourself	1 (6.7)	1/15 (6.7)	0.33	0.008–12.42	0.551
	No sign	14 (93.3)	13/15 (86.7)			
Herd and health management practices						
Presence of sheep	Yes	2 (13.3)	2/15 (13.3)	0.60	0.18–19.41	0.773
	No	13 (86.7)	12/15 (80.0)			
Presence of beef	Yes	5 (33.3)	4/15 (26.7)	0.14	0.004–4.22	0.260
	No	10 (66.7)	10/15 (66.7)			
Presence of buffalo	Yes	2 (13.3)	2/15 (13.3)	0.60	0.01–19.41	0.773
	No	13 (86.7)	12/15 (80.0)			
Presence of dog	Yes	12 (80.0)	11/15 (73.3)	1.09	0.03–33.38	0.958
	No	3 (20.0)	3/15 (20.0)			
Present of cat	Yes	3 (20.0)	3/15 (20.0)	0.91	0.03–27.82	0.958
	No	12 (80.0)	11/15 (73.3)			
Presence of rodent	Yes	9 (60.0)	9/15 (60.0)	5.18	0.17–150.46	0.338
	No	6 (40.0)	5/15 (33.3)			
Presence of bird	Yes	13 (86.7)	12/15 (80.0)	1.66	0.05–53.92	0.773
	No	2 (13.3)	2/15 (13.3)			
Presence of dense grass cover	Yes	5 (33.3)	4/15 (26.7)	0.14	0.004–4.22	0.260
	No	10 (66.7)	10/15 (66.7)			
Presence of unmanaged animal waste	Yes	1 (6.7)	1/15 (6.7)	0.33	0.008–12.42	0.551
	No	14 (93.3)	13/15 (86.7)			
Close to natural water resource	Yes	7 (46.7)	7/15 (46.7)	3.00	0.10–86.09	0.521
	No	8 (53.3)	7/15 (46.7)			
Type of pasture	Communal	1 (6.7)	1/15 (6.7)	0.33	0.008–12.42	0.551
	Private	14 (93.3)	13/15 (86.7)			
Other livestock in pasture	Mixed species	8 (53.3)	7/15 (46.7)	0.33	0.01–9.56	0.521
	Goat only	7 (46.7)	7/15 (46.7)			
Rotation of pasture	No	15 (100.0)	14/15 (93.3)	9.66	0.13–688.10	0.297
	Yes	0 (0.0)	0/15 (0.0)			
Neighboring (beef) 500 m	Yes	6 (40.0)	6/15 (40.0)	2.29	0.07–66.02	0.628
	No	9 (60.0)	8/15 (53.3)			
Introduced a new animal into herd within the last 2 years	Yes	10 (66.7)	10/15 (66.7)	7.00	0.23–206.79	0.260
	No	5 (33.3)	4/15 (26.7)			
Quarantine a new animal for at least 14 days	No	12 (80.0)	11/15 (73.3)	1.10	0.03–33.38	0.958
	Yes	3 (20.0)	3/15 (20.0)			
Blood sucking signs on skin	Presence	2 (13.3)	2/15 (13.3)	1.00	0.01–73.26	1.000
	No presence	2 (13.3)	2/15 (13.3)			
Repeated use of needles for drug administration	Yes	4 (26.7)	4/15 (26.7)	1.28	0.04–37.98	0.884
	No	11 (73.3)	10/15 (66.7)			
Annual hoof trimming	Yes	1 (6.7)	1/15 (6.7)	0.33	0.008–12.42	0.551
	No	14 (93.3)	13/15 (86.7)			
Sterile hoof trimmer before use	No	1 (6.7)	1/15 (6.7)	0.33	0.008–12.42	0.551
	Yes/Never trim	14 (93.3)	13/15 (86.7)			
Castrate buck	Yes	1 (6.7)	1/15 (6.7)	0.33	0.008–12.42	0.551
	No	14 (93.3)	13/15 (86.7)			
Sterilize castrate equipment before use	No	1 (6.7)	1/15 (6.7)	0.33	0.008–12.42	0.551
	Never castrate	14 (93.3)	13/15 (86.7)			
Breeder type	Natural mating	15 (100.0)	14/15 (93.3)	9.66	0.13–688.10	0.297
	Artificial insemination	0 (0.0)	0/15 (0.0)			
How to introduce a buck into a herd	Brought the used buck from another farm	7 (46.7)	6/15 (40.0)	0.25	0.008–7.33	0.425
	Brought the offspring buck	8 (53.3)	8/15 (53.3)			
Buck circulation between the community herds	Yes	7 (46.7)	7/15 (46.7)	3.00	0.10–86.09	0.521
	No	8 (53.3)	7/15 (46.7)			
Frequency of cleaning stable	Other	10 (66.7)	10/15 (66.7)	7.00	0.23–206.79	0.260
	Everyday	5 (33.3)	4/15 (26.7)			
Frequency of cleaning ground floor	Other	6 (40.0)	6/15 (40.0)	2.29	0.07–66.02	0.628
	Every week	9 (60.0)	8/15 (53.3)			
Insect control	No	9 (60.0)	8/15 (53.3)	1.30	0.02–75.12	0.896
	Yes	6 (40.0)	6/15 (40.0)			
Presence of vectors on the farm						
Presence of tick	Yes	3 (20.0)	3/15 (20.0)	0.91	0.03–27.82	0.958
	No	12 (80.0)	11/15 (73.3)			
Presence of stable flies	Yes	7 (46.7)	7/15 (46.7)	3.00	0.10–86.09	0.521
	No	8 (53.3)	7/15 (46.7)			
Presence of mosquitoes	Yes	15 (100.0)	14/15 (93.3)	9.66	0.13–688.10	0.297
	No	0 (0.0)	0/15 (0.0)			
Presence of Tabanus	Yes	6 (40.0)	5/15 (33.3)	0.19	0.006–5.60	0.338
	No	9 (60.0)	9/15 (60.0)			
Mixed-species Livestock farming						
Beef in farm	Yes	5 (33.3)	4/15 (26.7)	0.14	0.004–4.22	0.260
	No	10 (66.7)	10/15 (66.7)			
Buffalo in farm	Yes	3 (20.0)	3/15 (20.0)	0.91	0.03–27.82	0.958
	No	12 (80.0)	11/15 (73.3)			
Poultry in farm	Yes	7 (46.7)	6/15 (40.0)	0.25	0.008–7.33	0.425
	No	8 (53.3)	8/15 (53.3)			

Prevalence (Prev.), odd ratio (OR), and 95% confidence interval (95% CI) of the variable from 14 positive herds (93.33%) based on a total sample of 15 herds.

**Table 9:** Univariate analysis of individual-level risk factors for hemotropic mycoplasma (HM) infections in meat goat herds in Northeastern Thailand.

Risk Factor	Category	No. of Farms *	Total (%)	Prev. (%)	Crude OR	95% CI	*p*-Value
Gender	Female	14	78 (89.7)	48/87 (55.2)	2.00	0.49–8.04	0.329
	Male	6	9 (10.3)	4/87 (4.6)			
Age (years)	≥1	10	70 (80.4)	48/87 (55.2)	***7.09***	***2.07–24.23***	***0.002***
	<1	14	17 (19.6)	4/87 (4.6)			
BCS	≥3.5	15	81 (93.1)	52/87 (59.8)	***23.14***	***1.25–425.38***	***0.035***
	≤3	4	6 (6.9)	0/87 (0)			
FAMACHA score	≥3.5	13	79 (90.8)	49/87 (56.3)	2.72	0.60–12.22	0.191
	≤3	6	8 (9.2)	3/87 (3.4)			
PCV (%)	<22	11	71 (91.0)	42/87 (48.3)	0.86	0.28–2.65	0.805
	≥22	9	16 (9.0)	10/87 (11.5)			
Total protein (g/dL)	Not done	1	1 (1.1)	0/87 **			
	<6.2	12	58 (66.7)	32/87 (36.8)	0.49	0.18–1.29	0.152
	≥6.2	14	28 (32.2)	20/87 (23.0)			
Nematode (EPG)	Not done	1	11 (12.6)	9/87 (10.3)			
	0–500	12	54 (62.1)	34/87 (39.1)			
	>501	7	22 (25.3)	9/87 (10.3)	0.40	0.14–1.12	0.082

Prevalence (Prev.), odd ratio (OR), and 95% confidence interval (95% CI) of the variable from positive PCR assay (52/87; 59.77%) based on a total sample of 87 samples. * Number of farms contributing to the total number of animals in each category. ** not included in the univariate analysis.

**Table 10:** Multivariable Analysis of Individual-Level Risk Factors Associated with HM Among Meat Goat Herds in Northeast Thailand.

Risk Factor	Category	Adjusted OR	95% CI	*p*-Value
Gender	Female	3.07	0.36–25.84	0.302
	Male			
Age (years)	≥1	***9.88***	***1.73–56.48***	***0.010***
	<1			
BCS	≥3.5	1.46	0.56–3.81	0.430
	≤3			
FAMACHA score	≥3.5	1.15	0.49–2.70	0.734
	≤3			
PCV (%)	<22	1.26	0.22–7.00	0.788
	≥22			
Total protein (g/dL)	<6.2	0.67	0.19–2.37	0.54
	≥6.2			
Nematode (EPG; Eggs per gram)	0–500			
	>501	0.99	0.99–1.00	0.051

## Data Availability

The data that support the findings of this study are available from the corresponding author upon reasonable request.
